# Valgus Malalignment Due to Internally Malrotated Trochanteric Nail Placement, with Rotational Malalignment in Femoral Shaft Segmental Fracture Fixation, an Underestimated Avoidable Technical Error: A Case Report

**DOI:** 10.5704/MOJ.2003.011

**Published:** 2020-03

**Authors:** PX Hwang, NA Anuwar, YC Khaw, D Hadizie

**Affiliations:** Department of Orthopaedics, Universiti Sains Malaysia, Kubang Kerian, Malaysia

**Keywords:** valgus, nail, malalignment, Malunion, malrotated

## Abstract

Coronal malalignment due to malrotated trochanteric nail placement in femoral fracture fixation has never been reported. We present a case of a femoral segmental fracture fixed with a trochanteric nail, with a malrotated placement resulting in a valgus malaligned nail and femur, associated with a rotational malalignment. Knowledge of the modern nail design with proper intra-operative precautions, would avoid this underestimated technical error.

## Introduction

Fixation of a segmental femur fracture with interlocking nail is technically challenging. Great care is needed to prevent malalignment and malrotation. There are literature reviews which described malrotation and malalignment due to inadequate reduction^1^. There is however, no literature which has described malrotated femoral intramedullary nail placement leading could lead to valgus malalignment, a likely under-reported avoidable technical error. We present a case of femoral shaft segmental fracture fixed with a trochanteric nail complicated with a valgus malalignment as a result of an internal rotated placement, associated with rotational malalignment.

## Case Report

**Fig. 1:**
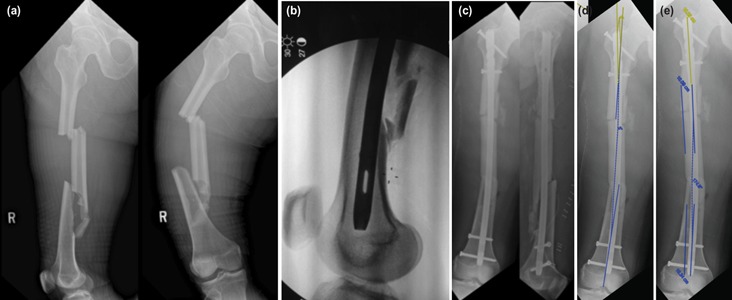
(a) Preop radiograph of right femoral segmental fracture, (b) Intraop fluoroscopic lateral knee view: two distal screws are parallel with lateral knee axis, (c) immediate post op radiograph anterior posterior view and (d) lateral view, (e) 5° valgus malalignment between proximal and distal femoral segments, 5.4° valgus malalignment between middle and lower third portion of the A2FN nail.

A 31 years old gentleman, had a road traffic injury with head on collision of his motorcycle with a car. He sustained a right femoral shaft segmental closed fracture (Fig. 1a), left tibial midshaft opened grade IIIa fracture and right humeral midshaft closed fracture. Within 24 hours, he underwent emergency damaged controlled surgery with left leg debridement and external fixation, right humeral fracture was splinted, while right femur was immobilised with skeletal traction.

Definitive fixation of right femoral and right humeral fractures were delayed due to left leg wound care. One-month post trauma, he underwent right humeral open reduction and plating, as well as right femoral open reduction and interlocking nail. Right femur was fixed with Synthes A2FN ^™^ (right, cannulated, diameter 10mm, length 380mm). Open reduction was necessary due to the formation of callus around fractures sites. Traction table was applied, posterolateral approach was utilised to access the fractures, callus was removed and open reduction of the fractures achieved. Appropriate trochanteric entry point was made, guide wire inserted and its tip was ensured to be in the centre before reaming. The nail was introduced with the insertion handle directed anteriorly followed by laterally after passage through the first fracture level. The femoral neck axis and the lateral knee axis were checked fluoroscopically to restore the rotational alignment to approximately 20°. A fluoroscopic true lateral knee view was obtained and the insertion handle was adjusted to allow ‘perfect circle’ of the two distal static screw holes for distal static screw placement (Fig. 1b). At this position the distal static screw holes and insertion handle were parallel to the true lateral knee axis. Two proximal static screws were subsequently inserted via targeting device from insertion handle.

Immediate post-operative radiograph was taken (Fig. 1c). There was 5° valgus malalignment between proximal and distal femoral segments in true anteroposterior femoral radiograph (as shown in Fig. 1d with patella located centrally). The middle and lower third portion of the nail was 5.4° in valgus (Fig. 1e), suggesting possibly the nail was in improper rotational placement.

**Fig. 2:**
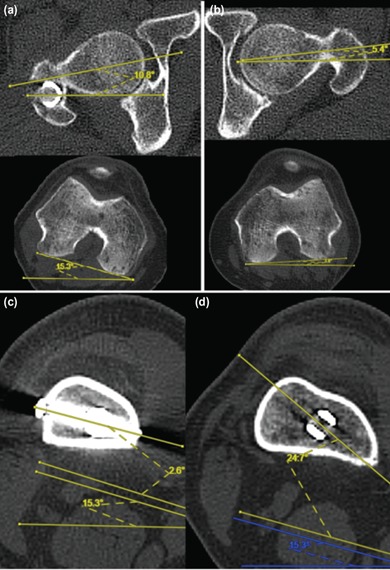
CT scan demonstrated right femur had external malrotation, and the nail placement was excessively internal rotated. (a) Right femur had 26.1° anteversion (10.8 + 15.3), (b) uninjured left femur had 1.6° retroversion (-5.4 + 3.8), (c) axial cut of right distal femur at level of static screw hole showed that the static screw was parallel to transcondylar axis, (d) axial cut of right distal femur at level of dynamic screw holes showed that the dynamic screw hole was 25° internal rotated in relation to transcondylar axis.

Computed tomography (CT) scan was done to determine rotational alignment of femur and nail rotational orientation. By measuring the differences between femoral neck axis and transcondylar axis, the right femur had 26.1° anteversion (Fig. 2a), while uninjured left femur had 1.6° retroversion (Fig. 2b). The distal static screw hole was parallel to transcondylar plane (Fig. 2c) while distal dynamic screw hole was 25° internal rotated in relation to transcondylar plane (Fig. 2d).

**Fig. 3:**
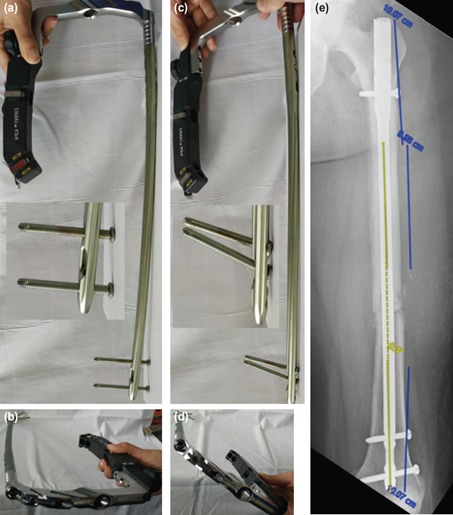
A2FN assembled to insertion handle, coronal alignments of the nail in relation to distal screws holes and insertion handle were shown. (a) When distal static screws holes are in true lateral plane, the nail is valgus, and (b) the insertion handle is horizontal. (c) When distal dynamic screw hole is in true lateral plane, the nail is straight, and (d) the insertion handle is anteverted (approximately 30°). (e) An example of femoral radiograph with A2FN shows that the lower two third of nail is straight when distal dynamic hole is well visualised.

To determine if the nail has appropriate design, the nail coronal alignment was demonstrated in relation to distal static and dynamic screw holes. The nail is valgus when the insertion handle is directly lateral, and the distal static screw holes are perpendicular to frontal plane (Fig. 3a, b). The nail is straight when the insertion handle is in approximately 30° anteversion and the distal dynamic holes are perpendicular to frontal plane (Fig 3c, d). A radiograph example confirms the nail is straight when distal dynamic screw hole is well visualised (Fig. 3e).

## Discussion

Fixation of the femoral segmental fracture with interlocking nail is prone to coronal, sagittal and rotational malalignment. Our case was complicated with both external malrotation and valgus malalignment. Despite attempt to restore femoral anteversion by using fluoroscopic technique, the external malrotation error can be due to rotational displacement of femur during insertion handle adjustment to get ‘full moon’, and failure to use uninjured side as baseline. Interestingly, an experiment by Suthersan *et al*^2^ proposed that during nail insertion, the distal femoral segment will be gripped and rotated by the nail’s spiral groove, causing malrotation. In our case however, it is more likely due to above mentioned technical error.

Angular malalignment is defined as more than 5° in coronal or sagittal plane and may lead to degenerative arthropathy^3^. Proximal and distal third shaft fractures are associated with higher incidence of coronal malalignment^1^. Other causes leading to coronal malalignment include improper entry point of trochanteric nail, inadequate reduction of the fracture, deforming forces of the muscles acting on the bone segments, spacious canal of proximal and third fractures at metaphyseodiaphyseal junction^4, 5^.

These reasons were not applicable to our case because attention had been given to ensure proper nail entry point, anatomical reduction, and central position of distal nail tip. The nail was excessively internally rotated, with the resulting manifestation of the designated anterior bow in valgus angulation, causing the valgus in the femur. The nail is designed to have no coronal bend in its lower two third if it is properly placed in designated rotation, which is perpendicular to distal dynamic holes, as shown above.

The Synthes Expert A2FN surgical technique brochure instructs to direct the insertion handle anteriorly during initial nail insertion, followed by gradual 90° lateral turn during the last one third of nail insertion. From Fig 3b, the insertion handle is supposed to be at anteverted position rather than 90° turn, otherwise it will be excessively internal rotated.

Interestingly, to our best knowledge coronal malalignment due to malrotated placement of interlocking nail has never been reported. Modern nail design consists of proximal lateral bend for trochanteric entry and anterior femoral curve for anterior femoral bow. This complex three-dimension curvature confuses the surgeon resulted this error to be underestimated. Orientating curves and direction of screw by direct visualising the implant before nail insertion, fluoroscopic assessment of distal screw holes direction are the techniques that can be used to avoid malrotation placement of nail.

In conclusion, trochanteric entry nail with malrotated placement could cause significant valgus malalignment. Good understanding of the nail design and proper intra-operative precautions are important to prevent this technical error.

## References

[1] Ricci WM, Bellabarba C, Lewis R, Evanoff B, Herscovici D, Dipasquale T (2001). Angular malalignment after intramedullary nailing of femoral shaft fractures.. J Orthop Trauma..

[2] Suthersan M, Harris I, Suzuki A (2012). Malrotation Due to a Design Element of a New Antegrade Femoral Nail.. Internet J Orthop Surg..

[3] Tetsworth K, Paley D. Malalignment, degenerative arthropathy. (1994). Orthop Clin North Am..

[4] Yun HH, Oh CH, Yi JW (2013). Subtrochanteric femoral fracture during trochanteric nailing for the treatment of femoral shaft fracture.. Clin Orthop Surg..

[5] Sims SH (2002). Subtrochanteric femur fractures.. Orthop Clin North Am..

